# Analgesic Efficacy of Low‐Level Laser Therapy for Postoperative Endodontic Pain: A Systematic Review and Meta‐Analysis

**DOI:** 10.1111/aej.70052

**Published:** 2026-01-06

**Authors:** Gabriela Bonacina, Rafael Chies Hartmann, Maximiliano Schünke Gomes, Daiana Elisabeth Böttcher

**Affiliations:** ^1^ Graduate Program in Dentistry, School of Health and Life Sciences Pontifical Catholic University of Rio Grande do Sul Porto Alegre Rio Grande do Sul Brazil; ^2^ Institute of Health Sciences, Universidade Feevale Novo Hamburgo Rio Grande do Sul Brazil; ^3^ Medical and Dental Center of the Military Police of Rio Grande do Sul Porto Alegre Rio Grande do Sul Brazil

**Keywords:** clinical, endodontics, low level laser therapy, nonsteroidal anti‐inflammatory drugs, postoperative pain

## Abstract

This systematic review analysed the analgesic efficacy of low‐level laser therapy (LLLT) compared to conventional drug therapy and placebo for postoperative pain in endodontics. PubMed, PubMed Central, Scopus, LILACS, SciELO, Virtual Health Library, Embase and Open Gray were searched. Clinical trials assessing pain intensity were included. Risk of bias was assessed using the RoB 2.0 tool. Nine studies were included, five classified as having low risk of bias. Eight studies demonstrated that LLLT was more effective in modulating postoperative pain compared to control interventions. Meta‐analysis of five studies (*I* ^2^ = 31%) showed statistically significant pain reduction with LLLT in comparison with placebo after 1 day (VAS mean difference = −0.56; 95% CI = [−0.74 to −0.38]; *p* < 0.001). LLLT may improve postoperative pain control after endodontic treatment. Additional high‐quality studies are necessary to strengthen the evidence base for LLLT's analgesic efficacy in endodontic applications.

## 1 Introduction

Postoperative pain following endodontic treatment (POEP) is a significant clinical concern, with reported incidence rates ranging from 2.5% to 69% [1, 2]. This pain arises from periapical inflammation triggered by mechanical, chemical or microbial factors, regardless of the endodontic technique employed [3, 4]. However, no demographic, medical or dental factors have been found to reliably predict moderate‐to‐severe postoperative pain after root canal treatment in previous observational studies [5].

The pathophysiology of POEP involves inflammatory mediators that increase vascular permeability and cellular chemotaxis [6], with apical extrusion of debris potentially exacerbating periapical inflammation [7]. Current pharmacological management primarily relies on analgesic and anti‐inflammatory drugs [8]. Conventional therapies, including acetaminophen, nonsteroidal anti‐inflammatory (NSAIDs) and opioids, are associated with significant adverse effects such as hepatotoxicity, gastrointestinal complications, renal impairment and risk of dependence or addiction [9, 10].

To address these limitations, new methods have been proposed to reduce POEP and improve patient quality of life and comfort. Photobiomodulation (PBM) has emerged as a promising nonpharmacological alternative, demonstrating analgesic, anti‐inflammatory and tissue regenerative properties without reported adverse effects, owing to its localised action [11, 12]. First introduced by Mester in 1967, low‐level laser therapy (LLLT) utilises specific light parameters (600–1000 nm wavelength; ≤ 500 mW output) to induce clinically significant biostimulation without thermal tissue effects [13, 14]. While its exact therapeutic mechanism remains unclear, LLLT appears to modulate inflammatory cascades through multiple pathways [15].

At the cellular level, LLLT promotes the resolution of acute‐phase inflammation by stimulating the cellular respiratory chain. It enhances mitochondrial respiratory chain activity, primarily through the activation of cytochrome C oxidase [13, 16]. This photobiomodulation process increases ATP synthesis [13, 17] and reduces inflammatory reaction induced by LPS [18]. Additionally, evidence suggests LLLT may inhibit nociception by reducing conduction velocity in Aδ and C nerve fibres, thereby reducing acute pain [19].

Despite these advances, systematic evaluations of PBM for endodontic pain management remain limited [20, 21, 22], particularly regarding comprehensive comparisons with conventional pharmacotherapy across different preoperative endodontic diagnoses. Therefore, this systematic review aims to analyse the available evidence regarding the analgesic efficacy of LLLT and compare its effectiveness with standard pharmacological treatments for managing postoperative pain in endodontics, providing evidence‐based recommendations for clinical practice.

## 2 Materials and Methods

This systematic review adhered to the Preferred Reporting Items for Systematic Reviews and Meta‐Analyses (PRISMA 2020) guidelines [23] and was registered in PROSPERO (CRD42023421521).

### 2.1 Literature Search Strategy

Two independent reviewers (GB and RCH) performed a comprehensive search across multiple databases including PubMed/MEDLINE, PubMed Central, Scopus, LILACS, SciELO, Virtual Health Library, Embase and OpenGrey (grey literature). The search strategy incorporated Medical Subject Headings (MeSH) terms and text words (tw.) related to LLLT and postoperative pain management, combined with Boolean operators (AND/OR). The complete search syntax is provided in Appendix S1.

The search included clinical trials and observational studies without restrictions on publication language or date, with the final search completed on January 13, 2025. Identified records were imported into EndNote X8 (Thompson Reuters, Toronto, Canada) for duplicate removal using both automated and manual methods. Manual searches of reference lists from included articles were also conducted to identify additional relevant studies.

### 2.2 Eligibility Criteria

Study selection followed a two‐phase screening process: initial title/abstract screening followed by full‐text evaluation against predefined criteria based on the PICOS framework [24], P patients undergoing root canal treatment, I Low‐Level Laser Therapy, C analgesic/anti‐inflammatory medication, or placebo, O postoperative endodontic pain levels S clinical trials. The treatment of interest was LLLT, which was compared to analgesic/anti‐inflammatory medication, or placebo. The primary outcome was postoperative pain levels following root canal treatment appointments. The study design included clinical trials.

Articles that did not meet the inclusion criteria or were review articles, case reports, case series or expert opinion were excluded.

### 2.3 Study Selection and Data Extraction

Two reviewers (GB and RCH) independently screened titles/abstracts and evaluated full texts for eligibility. Data were extracted using a standardised form, including: author(s), year of publication, country, study design, sample size, endodontic diagnosis, compared groups, treatment particularities that could influence postoperative pain, drug administration, drug administration protocol, laser device, laser application protocol, type of pain scale, intensity of postoperative pain after root canal treatment visit, time of pain measurement after visit and main results. All extracted data underwent cross‐verification to ensure accuracy. Discrepancies were resolved through discussion or consultation with a third reviewer (DEB).

### 2.4 Main Outcome Variable

The primary outcome was postoperative pain intensity following root canal treatment, assessed using validated pain scales: visual analogue scale (VAS), verbal rating scale (VRS) or numeric rating scale (NRS). For meta‐analysis, the outcome variable was grouped according to the time of pain measurement common to all included studies (1 day–24 h). NRS and VRS scores were converted to VAS equivalents based on established scale equivalencies [5, 25].

### 2.5 Quality Assessment

Risk of bias was evaluated using the Cochrane RoB 2.0 tool [24] by two independent reviewers (GB, RCH). The addressed domains were randomisation process, deviations from the intended intervention, missing outcome data, measurement of the outcome and selection of the reported result. Studies were classified as low, moderate or high risk of bias. Unclear cases were resolved by contacting authors by email at least three times to obtain additional information. Unresolved cases retained ‘unclear’ designation. Final risk classification was determined as follows: Studies that showed low risk in all domains were classified as having a low risk of bias; studies that raised concerns in at least one domain were classified as having a moderate risk of bias, and studies that presented at least one domain in the high‐risk range were classified as having a high risk of bias.

### 2.6 Strength of Evidence

The GRADE methodology [24] was applied using GRADEpro GDT (McMaster University, 2015) to assess evidence certainty, considering study limitations, consistency, directness, precision and publication bias.

### 2.7 Data Synthesis

Qualitative data were systematically organised and managed using Microsoft Excel (Microsoft Excel for MAC, Version 16.100, 2025). For quantitative synthesis, studies with comparable methodologies and complete data sets were included. The primary outcome was the effect of LLLT on postoperative pain control, with pain levels quantified at the 1‐day (24 h) postoperative interval. Continuous outcomes (VAS scores) were expressed as standard mean difference (SD) with a 95% confidence interval (CI). Subgroup analyses were performed by diagnostic category (irreversible pulpitis, necrotic pulp and apical periodontitis). Meta‐analysis was conducted using Review Manager 5.4 (The Cochrane Collaboration, Copenhagen, Denmark). Heterogeneity was assessed via the *I* ^2^ statistic, defined as low (*I* ^2^ ≤ 25%), moderate (*I* ^2^ > 25% and < 75%) and high (*I* ^2^ ≥ 75%) [24]. Meta‐analysis using the fixed effect model was performed when heterogeneity was ≤ 50%.

## 3 Results

### 3.1 Study Selection

**FIGURE 1:**
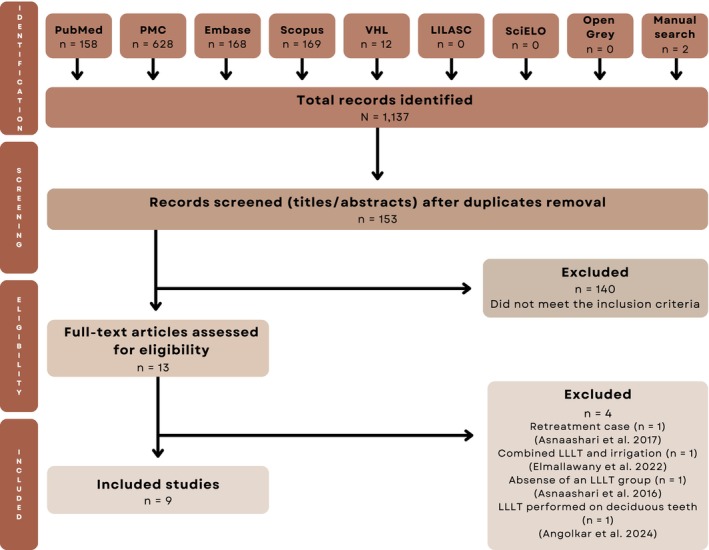
PRISMA flowchart.

Figure 1 presents the PRISMA flow diagram of the literature search and study selection process.

The electronic search yielded 1137 studies. After duplicate removal, 153 records underwent title and abstract screening. Thirteen articles were selected for full‐text assessment [14, 26, 27, 28, 29, 30, 31, 32, 33, 34, 35, 36, 37]. Nine studies met the inclusion criteria [26, 27, 28, 29, 30, 31, 35, 36, 37], while four were excluded [14, 32, 33, 34].

### 3.2 Overall Characteristics of the Included Studies

The characteristics of the included studies are summarised in Table 1. All nine studies were randomised clinical trials published between 2018 and 2023 [26, 27, 28, 29, 30, 31, 35, 36, 37]. The studies originated from Brazil [26, 28, 30], Turkey [27, 31, 35], Iran [29] and India [37].

**TABLE 1:** Overall characteristics of the included studies (*N* = 9).

Study	Country	Design	*N*	Diagnosis	Groups	*n*	Pain intensity scale
Guimarães et al. 2021	Brazil	RCT	70	Asymptomatic apical periodontitis	Control—no laser Test—laser (aPDT + LLLT)	35	VAS
Ismail et al. 2023	Egypt	RCT	180	Acute or chronic symptomatic apical periodontitis Symptomatic apical periodontitis Cohen's classification	Group 1—LLLT Group 2—LAI Group 3—mock laser (ML)		VAS
Kaplan et al. 2021	Turkey	RCT	60	Asymptomatic apical periodontitis	Control – no laser Test—laser	30	VAS
Lopes et al. 2019	Brazil	RCT	60	Symptomatic irreversible pulpitis	Control – no laser Test—laser	30	VRS NRS
Naseri et al. 2020	Iran	RCT	75	Symptomatic irreversible pulpitis	Control – no laser Test—BLI Test—BI	25	VAS
Nunes et al. 2020	Brazil	RCT	70	Symptomatic irreversible pulpitis	Control – Ibuprofen Test—laser	35	VRS NRS
Sağlam & Aladağ 2023	Turkey	RCT	80	Symptomatic irreversible pulpitis	Group 1—LLLT Placebo Group 2—LLLT Group Group 3—Ozone Placebo Group 4—Ozone Group		VAS
Shah et al. 2022	India	RCT	42	Apical periodontitis	Case group (I)—LLLT Control group (II)—no LLLT		VAS
Doğanay Yıldız & Arslan 2018	Turkey	RCT	42	Symptomatic apical periodontitis Cohen's classification	Control—no laser Placebo—mock laser Test—laser	14	VAS

Abbreviations: BI, buccal irradiation; BLI, buccal lingual irradiation; LAI, laser‐activated irrigation; *n*, subjects per group; *N*, total; NRS, numeric rating scale; RCT, randomized controlled trial; VAS, visual analogue scale; VRS, verbal rating scale.

Two trials evaluated the analgesic effect of LLLT in cases of asymptomatic apical periodontitis [26, 27], two evaluated symptomatic apical periodontitis cases described according to Cohen's classification [31, 36], three evaluated cases of symptomatic irreversible pulpitis [28, 29, 30], and one study included patients with unspecified apical periodontitis [37].

Sample size of the included studies ranged from 42 to 180 patients (mean: 70). One study evaluated the combined effect of LLLT and photodynamic therapy (PDT) in the intervention group [26]. Another study evaluated two intervention groups varying sites/times of laser application [29]. One study included both placebo (no laser) and control group (laser mock) [31]. Eight studies compared LLLT to nonmedicated controls [26, 27, 28, 29, 31, 35, 36, 37], and one study used anti‐inflammatory medication as a comparator [30].

Seven studies evaluated pain intensity through the VAS [26, 27, 29, 31, 35, 36, 37] and two used VRS and NRS [28, 30]. Pain was most frequently measured at 24 h (1 day) posttreatment, though assessment times varied across studies.

### 3.3 Technical Features Related to Endodontic Treatment Protocol

The included studies demonstrated variation in treatment protocols. Seven studies performed endodontic treatments in a single visit [26, 28, 29, 30, 31, 35, 36] while two studies employed a two‐visit approach [27, 37]. Foraminal enlargement was specifically reported in one study [29]. Regarding instrumentation techniques, five studies utilised reciprocating systems for root canal preparation [26, 28, 30, 31, 35], two studies used continuous rotation systems [27, 36], one study employed either a manual technique or continuous rotation depending on case indications [29], and one study exclusively used the step‐back manual technique [37].

### 3.4 Characteristics Related to Laser Application Protocol

The laser application protocols employed in the included studies are summarised in Table 2. All trials incorporated laser therapy in their test groups, with variations in laser type and parameters. Two studies utilised indium–gallium–aluminium (InGaAlP) lasers [28, 30], while one study employed a gallium–aluminium–arsenide (GaAlAs) laser [26]. The remaining six studies reported using diode lasers [26, 29, 31, 35, 36, 37]. The wavelengths applied ranged from 660 nm [37] to 980 nm [27, 31, 36]. The most frequently reported output power was 100 mW [26, 28, 29, 30, 36, 37]. However, two studies did not specify the energy dose delivered per cm^2^ [27, 31]. Application times varied across studies, ranging from 40 to 160 s.

**TABLE 2:** Laser application protocols in included studies (*N* = 9).

Study	Laser device	Wavelength (nm)	Power (mW)	Dose (J/cm^2^)	Fiber diameter (μm)	Application time (s)
Guimarães et al. 2021	GaA1As	808 ± 10	100	133	NR	40
Ismail et al. 2023	Diode	980	100	30	200	60
Kaplan et al. 2021	Diode	980	24 000	NR	200	40
Lopes et al. 2019	InGaAlP	808	1000	90	200	25
Naseri et al. 2020	Diode	808	100	260/590	600	80 (BI) 160 (BLI)
Nunes et al. 2020	InGaAlP	808	100	360	200	100
Sağlam & Aladağ 2023	Diode	970	50	15	200	30
Shah et al. 2022	Diode	660	100	6	NR	60
Doğanay Yıldız & Arslan 2018	Diode	970	5000	NR	200	60

Abbreviations: BI, buccal irradiation; BLI, buccal lingual irradiation; GaAlAs, Gallium–Aluminium–Arsenide; InGaAlP, Indium–Gallium–Aluminum; NR, not reported.

### 3.5 LLLT's Analgesic Capacity

The overall results of pain intensity across different groups and measurement times are summarised in Table 3, except for one study [31], which was excluded due to unreported standard deviation values. While one study [26] found no significant differences in postoperative pain between control and test groups at any evaluated time point, all other studies demonstrated statistically significant benefits of LLLT compared to placebo or medication groups [30, 31, 36, 37]. Regarding the time point evaluation, one study [27] reported significantly higher mean pain levels (*p* < 0.05) in controls at 24 h post‐first treatment and at 24–48 h after the second visit. Another study [29] found the group receiving 160 s of laser application had a significantly lower incidence of postoperative pain than controls at all intervals. Conversely, a third study [28] observed that LLLT effectively controlled pain at 6 h (*p* = 0.04) and 24 h (*p* = 0.02) posttreatment, though not at 12 h (*p* > 0.05).

**TABLE 3:** Postoperative pain intensity outcomes by treatment group and timepoint (*N* = 8).

Study	Time	Pain intensity scale	Group	Pain intensity, mean (SD)
Guimarães et al. (2021), *N* = 70, *n* = 35	1 day	VAS	Control	0.97 (1.88)
			LLLT	0.42 (1.11)
	2 days	VAS	Control	0.62 (1.69)
			LLLT	0.51 (1.35)
	3 days	VAS	Control	0.71 (1.94)
			LLLT	0.48 (1.48)
	4 days	VAS	Control	0.48 (1.46)
			LLLT	0.11 (0.32)
	5 days	VAS	Control	0.35 (1.08)
			LLLT	0.11 (0.40)
	6 days	VAS	Control	0.25 (0.74)
			LLLT	0.02 (0.16)
	7 days	VAS	Control	0.11 (0.40)
			LLLT	0.02 (0.16)
	14 days	VAS	Control	0.25 (1.06)
			LLLT	0.00 (0.00)
	30 days	VAS	Control	0.05 (0.33)
			LLLT	0.00 (0.00)
Ismail et al. (2023), *N* = 180, *n* = 60	1 day	VAS	Control	4.80 (2.30)
			LLLT	2.00 (1.00)
	2 days	VAS	Control	3.20 (1.80)
			LLLT	1.80 (1.00)
	3 days	VAS	Control	1.60 (1.30)
			LLLT	1.10 (1.00)
Kaplan et al. (2021), *N* = 60, *n* = 30	8 h	VAS	Control (1st visit)	2.57 (2.85)
			LLLT (1st visit)	1.00 (1.02)
			Control (2nd visit)	0.57 (1.13)
			LLLT (2nd visit)	0.30 (0.65)
	1 day	VAS	Control (1st visit)	1.90 (2.30)
			LLLT (1st visit)	0.33 (0.92)
			Control (2nd visit)	0.30 (0.88)
			LLLT (2nd visit)	0.00 (0.00)
	2 days	VAS	Control (1st visit)	0.90 (1.84)
			LLLT (1st visit)	0.20 (0.61)
			Control (2nd visit)	0.27 (0.69)
			LLLT (2nd visit)	0.00 (0.00)
	7 days	VAS	Control (1st visit)	0.00 (0.00)
			LLLT (1st visit)	0.00 (0.00)
			Control (2nd visit)	0.00 (0.00)
			LLLT (2nd visit)	0.00 (0.00)
Lopes et al. (2019), *N* = 60, *n* = 30	6 h	VRS	Control	0.77 (0.82)
			LLLT	0.53 (0.86)
	12 h	VRS	Control	0.47 (0.63)
			LLLT	0.30 (0.70)
	1 day	VRS	Control	0.40 (0.62)
			LLLT	0.10 (0.40)
	6 h	NRS	Control	1.87 (2.65)
			LLLT	1.43 (2.60)
	12 h	NRS	Control	1.27 (2.18)
			LLLT	0.77 (1.98)
	1 day	NRS	Control	1.00 (2.08)
			LLLT	0.27 (1.05)
Naseri et al. (2020), *N* = 75, *n* = 25	0 h	VAS	Control	6.28 (1.79)
			LLLT (BI)	6.44 (1.53)
			LLLT (BLI)	6.36 (1.52)
	4 h	VAS	Control	4.56 (2.34)
			LLLT (BI)	4.24 (2.43)
			LLLT (BLI)	2.96 (2.15)
	8 h	VAS	Control	3.72 (2.15)
			LLLT (BI)	3.08 (2.36)
			LLLT (BLI)	1.16 (1.62)
	1 day	VAS	Control	1.92 (1.91)
			LLLT (BI)	1.16 (1.49)
			LLLT (BLI)	0.32 (0.85)
	2 days	VAS	Control	0.92 (1.65)
			LLLT (BI)	0.44 (0.87)
			LLLT (BLI)	0.16 (0.62)
Nunes et al. (2020), *N* = 70, *n* = 35	6 h	VRS	Control (Ibuprofen)	2.25 (0.15)
			LLLT	2.14 (0.14)
	12 h	VRS	Control (Ibuprofen)	0.74 (0.10)
			LLLT	0.34 (0.81)
	1 day	VRS	Control (Ibuprofen)	0.42 (0.85)
			LLLT	0.85 (0.48)
	3 days	VRS	Control (Ibuprofen)	0.28 (0.28)
			LLLT	0.00 (0.00)
	6 h	NRS	Control (Ibuprofen)	1.51 (0.21)
			LLLT	0.62 (0.15)
	12 h	NRS	Control (Ibuprofen)	1.11 (1.19)
			LLLT	0.40 (0.10)
	1 day	NRS	Control (Ibuprofen)	0.60 (0.12)
			LLLT	0.11 (0.60)
	3 days	NRS	Control (Ibuprofen)	0.02 (0.28)
			LLLT	0.00 (0.00)
Sağlam & Aladağ (2023), *N* = 80, *n* = 20	1 day	VAS	Control	28.40 (12.90)
			LLLT	20.50 (6.10)
	2 days	VAS	Control	27.90 (12.20)
			LLLT	13.30 (6.60)
	3 days	VAS	Control	25.00 (9.40)
			LLLT	8.80 (2.50)
	4 days	VAS	Control	19.30 (9.80)
			LLLT	10.00 (0.00)
	5 days	VAS	Control	14.20 (7.30)
			LLLT	15.20 (7.50)
	6 days	VAS	Control	12.10 (5.70)
			LLLT	15.70 (7.50)
	7 days	VAS	Control	15.00 (8.70)
			LLLT	12.30 (6.80)
Shah et al. (2022), *N* = 40, *n* = 20	0 days	VAS	Control	3.50 (3.05)
			LLLT	3.05 (3.01)
	7 days	VAS	Control	2.65 (2.23)
			LLLT	2.00 (1.94)
	14 days	VAS	Control	1.90 (1.68)
			LLLT	1.25 (1.68)

Abbreviations: 1 day, 24 h; LLLT, low level laser therapy; NRS, numeric ranking scale; SD, standard deviation; VAS, visual analogue scale; VRS, verbal rating scale.

### 3.6 Risk of Bias Assessment

**FIGURE 2:**
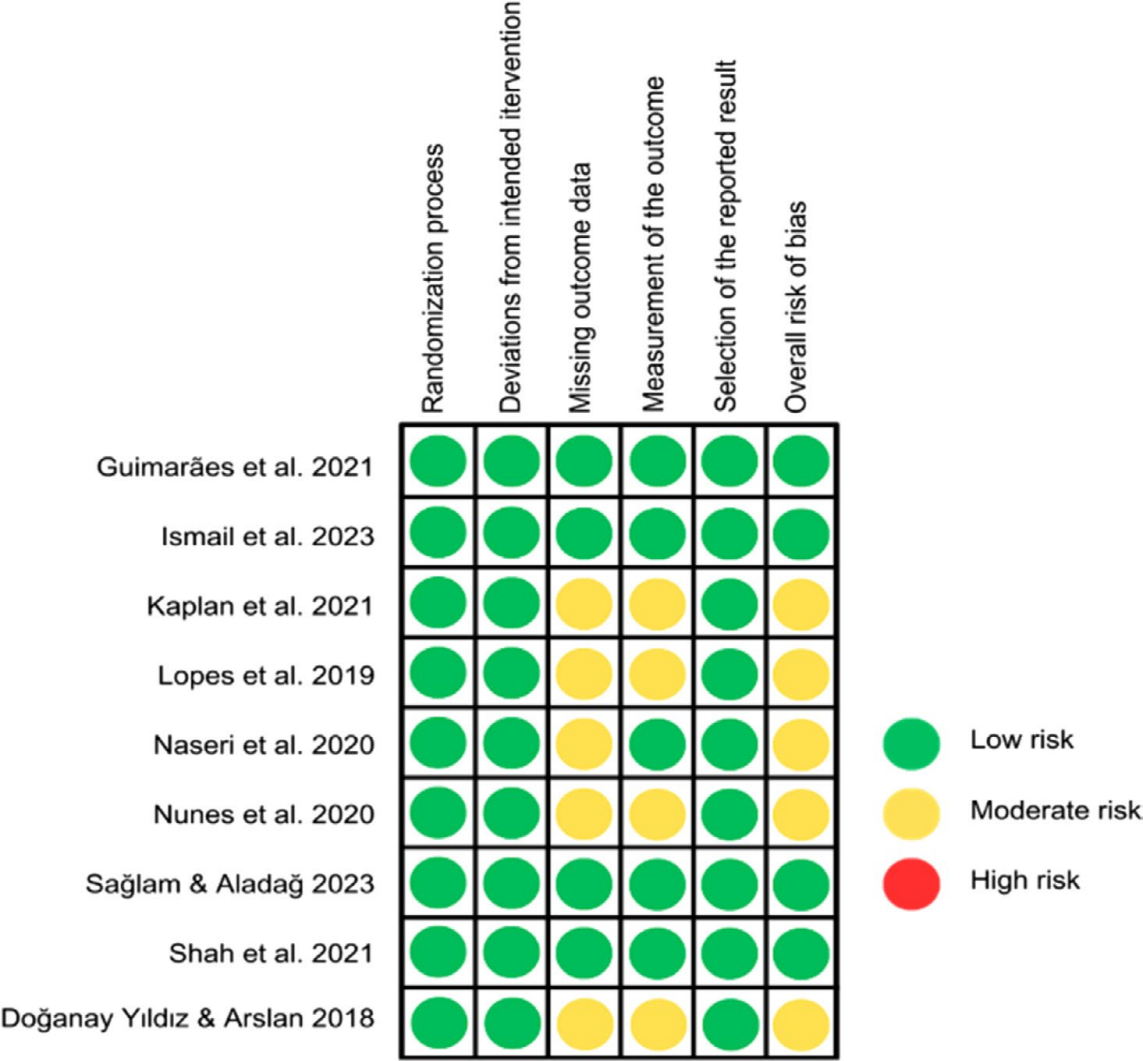
Risk of bias assessment.

The methodological quality of included studies was evaluated using the Cochrane Risk of Bias tool for randomised trials (RoB 2.0), with results presented in Figure 2. Among the nine included studies, four demonstrated low risk of bias [26, 35, 36, 37], while five were classified as moderate risk [27, 28, 29, 30, 31]. All studies adequately described their randomisation processes; however, four failed to report participant blinding procedures [27, 28, 30, 31] as well as losses [27, 28, 29, 30]. One study [31] omitted reporting standard deviation values. Notably, none of the studies exhibited high risk or concerns in the ‘selection of reported results’ domain.

### 3.7 Strength of Evidence

The certainty of evidence for all evaluated outcomes was systematically assessed using the GRADE methodology, with detailed results presented in Table 4.

**TABLE 4:** Certainty of the evidence from included studies according to the GRADE tool (*N* = 9).

Number of studies	9 RCT
Risk of bias	Serious^a^
Inconsistency	Serious^b^
Indirectness	Serious^c^
Imprecision	Not serious
Other considerations	None
Overall certainty of evidence	Moderate

Abbreviation: RCT, randomized controlled trial.

^a^ Five studies had a moderate overall risk of bias.

^b^ High heterogeneity (*I* ^2^ = 67%) among the apical periodontitis studies.

^c^ Significant differences in population or intervention characteristics.

The body of evidence was assessed as providing moderate certainty regarding LLLT's analgesic effects in postendodontic pain management. This determination reflects serious limitations in three key domains: risk of bias (due to methodological variations across studies), inconsistency (in treatment protocols and outcome measures) and indirectness (of population or intervention characteristics). However, the evidence demonstrated no serious imprecision in effect estimates, and no other modifying factors (such as publication bias or dose–response relationships) were identified that would alter this certainty rating.

### 3.8 Meta‐Analysis

A meta‐analysis was conducted incorporating five randomised clinical trials [26, 27, 28, 29, 30]. One eligible study [31] was excluded due to unavailable standard deviation values, despite attempts to contact the authors. Pain intensity, measured by visual analogue scale (VAS) at the 24‐h (1 day) postoperative time point, served as the primary outcome measure.

**FIGURE 3:**
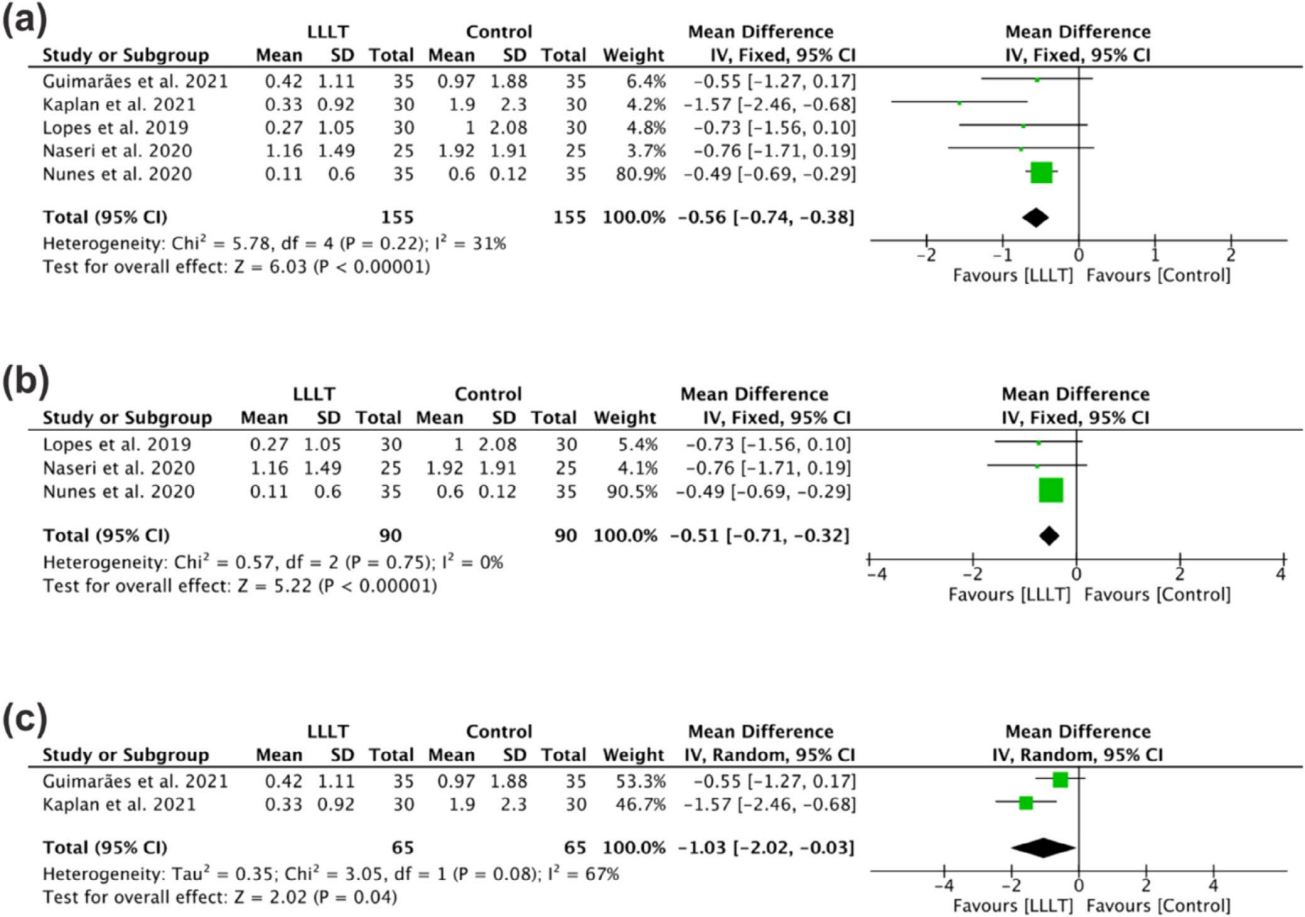
Forest plots of meta‐analysis comparing postoperative pain intensity between LLLT and control groups: (a) Overall analysis; (b) vital pulp subgroup; (c) asymptomatic apical periodontitis subgroup.

The forest plot analysis (Figure 3a) revealed statistically significant differences in analgesic efficacy between LLLT and placebo groups (*p* < 0.00001), with effect estimates favouring the LLLT intervention. The observed heterogeneity among studies was low (*I* ^2^ = 31%), indicating consistent treatment effects across the included trials.

Subgroup analyses were conducted based on preoperative pulp status (vital vs. necrotic). Figure 3b presents the forest plot for teeth diagnosed with pulpitis, demonstrating statistically significant differences in analgesic efficacy between LLLT and control groups (placebo/ibuprofen), favouring LLLT (*p* < 0.00001). The analysis showed perfect consistency among studies (*I* ^2^ = 0%).

For teeth with necrotic pulps (asymptomatic apical periodontitis), Figure 3c reveals that LLLT maintained statistically superior analgesic effects compared to placebo (*p* = 0.04), though with moderate heterogeneity (*I* ^2^ = 67%). Due to this variability, a random‐effects model was employed for this subgroup analysis.

## 4 Discussion

The management of postoperative endodontic pain remains a critical area of investigation in dentistry, with low‐level laser therapy emerging as a promising and effective nonpharmacological intervention. This systematic review synthesises evidence from nine randomised clinical trials, demonstrating that LLLT provides significant analgesic effects compared to placebo and NSAIDs. However, the overall certainty of evidence is moderate due to methodological limitations in the included studies. These findings align with recent evidence [20] but contrast with earlier reviews [21, 22], likely due to differences in study inclusion criteria and analytical approaches.

The GRADE assessment yielded moderate‐quality evidence, with the main limitations stemming from risks of bias in blinding procedures and substantial heterogeneity (*I* ^2^ = 67%) among studies involving necrotic pulp. These risks are directly illustrated by the inconsistent blinding methodologies observed across the included studies. Operator blinding was not universally applied; only three studies [28, 30, 36] employed the more rigorous method of postprocedural randomisation to prevent intraoperative bias. Patient blinding strategies were also variable and often inadequate, with most techniques being potentially detectable. In fact, true simulation of the laser application (using a protected tip to prevent light emission) was achieved in only two studies [26, 37]. Notably, despite these methodological concerns, the evidence demonstrated sufficient precision (*n* > 300 events; 95% CI) and did not require downgrading for publication bias, effect size, confounding or dose–response relationships, as outcome measures were standardised [25].

Postoperative pain represents a significant healthcare challenge, contributing to increased healthcare utilisation, decreased productivity and reduced quality of life [11]. The growing misuse of analgesic medications has also become a critical public health concern worldwide [38]. Our findings support LLLT's therapeutic potential in endodontic pain management, though the clinical relevance of the observed pain reduction requires careful interpretation due to modest effect sizes and evidence limitations. Nevertheless, LLLT represents a valuable nonpharmacological alternative that may help establish safer analgesic protocols and reduce dependency on potentially harmful substances.

Our study provides critical insights into how preoperative diagnosis influences LLLT efficacy. In vital teeth with pulpitis, conventional endodontic treatment alone typically alleviates symptoms by removing inflamed pulp tissue, except in advanced cases exhibiting periapical involvement or iatrogenic injury [39]. This observation suggests that reported LLLT benefits in pulpitis cases may be partially attributable to the removal of the inflammatory source rather than specific photobiomodulation effects.

Notably, our analysis confirms LLLT efficacy in vital pulp cases, with all four included studies demonstrating statistically significant pain reduction [28, 29, 30, 35]. These findings align with the inflammatory cascade in pulpitis, in which preoperative pain originates from neutrophil‐mediated acute inflammation beneath carious lesions [40]. The observed analgesic outcomes likely result from the combined effects of inflamed pulp removal and LLLT's modulation of neutrophil‐driven inflammatory responses. LLLT's effectiveness in mitigating mechanical trauma‐induced inflammation during endodontic procedures is particularly noteworthy.

In contrast, LLLT outcomes for necrotic pulp cases exhibited greater variability, with one of three studies showing no significant effects [26]. This inconsistency likely reflects fundamental pathophysiological differences, as pain mechanisms in nonvital teeth primarily involve persistent periapical inflammation and microbial factors rather than pulpal inflammation [41]. Our subgroup analysis provides crucial insights into these diagnosis‐dependent variations in LLLT efficacy. The therapeutic challenge in apical periodontitis differs substantially, characterised by persistent periapical inflammation requiring extended resolution time following microbial elimination [42]. Increased postoperative pain risk in these cases is attributed to two factors: potential extrusion of infected dentinal debris during canal instrumentation [7] and sustained periapical inflammatory responses [6]. These distinct pathological mechanisms necessitate careful interpretation when evaluating LLLT's therapeutic potential across diagnostic categories.

Recent systematic review evidence [20] suggests that adjunctive photodynamic therapy (PDT) could enhance postoperative endodontic pain management through improved antimicrobial efficacy. While PDT appears effective in reducing pain risk from microbial extrusion during instrumentation, its benefits for pre‐existing pain in symptomatic apical periodontitis remain unclear. The combined use of LLLT and PDT was evaluated, with no significant differences found compared to placebo despite variations in laser tip positioning [26].

Collectively, these findings suggest that preoperative pulp status may influence treatment outcomes more substantially than laser parameters alone, though further investigation is needed. The scarcity of studies investigating LLLT for symptomatic apical periodontitis and acute apical abscess likely stems from methodological challenges. The emergent nature of these conditions often precludes standardised randomisation, while spontaneous pain resolution before treatment compromises study feasibility. This represents a critical knowledge gap, as symptomatic apical periodontitis is one of the most clinically challenging scenarios for postoperative pain management. The current lack of robust data on these acute conditions impairs the development of optimised treatment protocols that could improve patient quality of life.

Variability in endodontic treatment protocols also presents challenges for quantitative analysis. Current evidence suggests that single‐visit versus multiple‐visit treatment does not significantly influence postoperative pain levels [43] or LLLT outcomes in the included studies. Foraminal enlargement, mentioned in four studies but implemented in only one, has been associated with increased 24‐h postoperative pain in necrotic teeth [44], though it did not alter LLLT's analgesic efficacy [26]. Furthermore, the same study [26] suggests that using PDT as an adjuvant therapy to reduce bacterial load should minimise bacterial extrusion and remnants, thereby decreasing perceived pain. This conclusion, however, is limited as the study lacked a control group to isolate PDT's specific effect on pain, using LLLT solely for comparative postoperative assessment. Despite variations in root canal preparation techniques, instrumentation methods did not appear to influence LLLT effectiveness, consistent with findings that alternative instruments do not significantly increase apical debris extrusion or postoperative pain [45]. Future studies should provide more comprehensive procedural details to elucidate interactions between endodontic techniques and LLLT analgesia.

It is noteworthy that all studies in this review originated from global south nations, introducing socioeconomic considerations. While the biopsychosocial model acknowledges socioeconomic influences on pain perception [46], the impact of sociodemographic factors, cultural background, educational attainment and healthcare disparities on endodontic pain remains incompletely understood [5]. These variables may substantially modify pain perception and treatment outcomes, warranting further investigation.

Accurate pain assessment is critical for managing endontic symptoms. While pain is a complex multidimensional experience, intensity measurement remains a critical component of therapeutic decision‐making. The included studies predominantly used three established self‐report instruments: Visual analogue scale (VAS), verbal rating scale (VRS) and numerical rating scale (NRS). The VAS demonstrates good sensitivity for detecting treatment effects but poses challenges for patients with perceptual‐motor impairments [47]. The VRS offers validated reliability [47] and patient preference but reduced sensitivity to clinical changes. Comparative analyses suggest both VAS and Numerical Rating Scale (NRS) demonstrate superior specificity for pain intensity [48], also justifying their use in most included trials. This subjectivity, compounded by variations in measurement timing and tools, contributes to significant heterogeneity in reported outcomes.

The meta‐analysis of five included studies focused on pain measurements 24 h postintervention, a standardised time point assessed across all studies. This time frame represents a clinically significant time point as approximately 40% of patients experience postoperative pain at this stage [4]. To ensure comparability, only single‐application LLLT groups were analysed. One study [29] included an additional group with LLLT applied at two points per root; this meta‐analysis considered only the single‐application group.

LLLT's therapeutic efficacy depends on technical parameters, including laser type, energy output (mW), energy density (J/cm^2^), wavelength (nm) and irradiation time [13]. All included studies used diode lasers, established for orofacial pain management [49]. While LLLT exhibits dose‐dependent effects, the optimal analgesic dosage remains undefined [21]. Seven studies employed doses within recommended ranges, but two failed to report this parameter. Wavelength selection is critical for tissue penetration, with the 670–900 nm range being most effective [13]. Four studies used wavelengths within this range, while others employed shorter (660 nm) [37] or longer wavelengths (970–980 nm) [27, 31, 35, 36] without apparent efficacy reduction. Inconsistent reporting of energy density parameters limits dose–response analysis, underscoring the need for standardised protocols.

Only one study [30] directly compared LLLT with pharmacological intervention, demonstrating LLLT's superior analgesic efficacy over ibuprofen at all evaluated time points (6 h: *p* < 0.001; 12 h: *p* = 0.005; 24 h: *p* < 0.001). While NSAIDs remain a mainstay for mild‐to‐moderate endodontic pain [50], their potential adverse effects highlight the need for nonpharmacological alternatives like LLLT.

In summary, our findings contrast with those of Alonaizan & Al Fawaz (2019), where most studies reported no significant differences in postendodontic pain after LLLT [22]. This discrepancy may stem from methodological differences. Our results align more closely with Luo et al. (2024), which found high‐quality evidence supporting adjunctive laser therapies for reducing postoperative endodontic pain [20]. These insights may guide optimised pain prevention strategies, but knowledge gaps persist regarding LLLT application in acute apical conditions. Future research should prioritise standardised reporting of laser parameters, enhanced blinding methods, multicentre trials, focused studies on acute apical periodontitis and cost‐effectiveness analyses.

The synthesis of current evidence from randomised clinical trials suggests that low‐level laser therapy (LLLT) demonstrates potential as an effective adjunct for postoperative pain management 24 h following endodontic treatment. However, the moderate quality of available evidence, primarily due to methodological limitations in existing studies, necessitates further high‐quality investigations with standardised protocols to establish definitive conclusions regarding LLLT's analgesic efficacy. Notably, comparative studies evaluating LLLT against conventional pharmacological approaches remain scarce, representing a critical gap in the current literature. As endodontics increasingly embraces minimally invasive strategies, LLLT emerges not only as a promising therapeutic modality but also as a valuable research framework for advancing our understanding of biomodulatory pain control mechanisms.

## Author Contributions

Each author has made substantial contributions to the study's conception, design, analysis and manuscript preparation. All authors have approved the final version of this submission.

## Conflicts of Interest

The authors declare no conflicts of interest.

## Supporting information


**Appendix S1:** aej70052‐sup‐0001‐Supinfo1.docx.

## Data Availability

Data sharing not applicable to this article as no datasets were generated or analysed during the current study.
