# Supplementary material for: Analgesic Efficacy of Low‐Level Laser Therapy for Postoperative Endodontic Pain: A Systematic Review and Meta‐Analysis
Source: Aust Endod J. 2026 Jan 6;52(1):310–22. doi: 10.1111/aej.70052 (PMC13051047; doi:10.1111/aej.70052)
Supplement: Supplementary file 1 — Appendix S1: aej70052‐sup‐0001‐Supinfo1.docx. [file AEJ-52-310-s001.docx]

**Supplementary Material 1** Search terms according to database

| PubMed/PubMed Central/Scopus | (((((((((((((((("low level laser therapy") OR ("cold laser")) OR ("low level laser irradiation")) OR ("laser therapy, low power")) OR ("laser therapy, low level")) OR ("photobiomodulation therapy")) OR ("laser phototherapy")) AND (endodontic)) OR (endodontics)) OR ("endodontic therapy")) OR ("endodontic treatment")) OR ("root canal treatment")) OR ("root canal therapy")) OR (pulpectomy)) OR (pulpitis)) OR ("apical periodontitis")) AND ("post operative pain") |
| --- | --- |
| LILACS/SciELO/VHL/Open Grey | (low level laser therapy) OR (cold laser) OR (low level laser irradiation) OR (laser therapy, low power) OR (laser therapy, low level) OR (photobiomodulation therapy) OR (laser phototherapy) AND (endodontic) OR (endodontics) OR (endodontic therapy) OR (endodontic treatment) OR (root canal treatment) OR (root canal therapy) OR (pulpectomy) OR (pulpitis) OR (apical periodontitis) AND (post operative pain) |
| Embase | ((**'low level laser therapy'**/exp OR **'low level laser therapy'** OR **'cold laser'** OR **'low level laser irradiation'** OR **'laser therapy, low power'** OR **'laser therapy, low level'**/exp OR **'laser therapy, low level'** OR **'photobiomodulation therapy'**/exp OR **'photobiomodulation therapy'** OR **'laser phototherapy'**) AND **endodontic** OR **'endodontics'**/exp OR **endodontics** OR **'endodontic therapy'** OR **'endodontic treatment'** OR **'root canal treatment'**/exp OR **'root canal treatment'** OR **'root canal therapy'**/exp OR **'root canal therapy'** OR **'pulpectomy'**/exp OR **pulpectomy** OR **'pulpitis'**/exp OR **pulpitis** OR **'apical periodontitis'**/exp OR **'apical periodontitis'**) AND **'post operative pain'** |
